# DNA Damage and Activation of cGAS/STING Pathway Induce Tumor Microenvironment Remodeling

**DOI:** 10.3389/fcell.2021.828657

**Published:** 2022-02-21

**Authors:** Rong Shen, Disheng Liu, Xiaoning Wang, Zhao Guo, Haonan Sun, Yanfeng Song, Degui Wang

**Affiliations:** ^1^ School of Basic Medical Sciences, Lanzhou University, Lanzhou, China; ^2^ The First Hospital of Lanzhou University, Lanzhou, China; ^3^ School of Medicine, Shandong University, Jinan, China

**Keywords:** DNA damage, cGAS/STING, interferon, immune response, TME, remodeling, oncotherapy

## Abstract

DNA damage occurs throughout tumorigenesis and development. The immunogenicity of DNA makes it an immune stimulatory molecule that initiates strong inflammatory responses. The cGAS/STING pathway has been investigated as a critical receptor in both exogenous and endogenous DNA sensing to activate the innate immune response. Growing lines of evidence have indicated that activation of the cGAS/STING pathway is critical in antitumor immunity. Recent studies have demonstrated the outstanding advancement of this pathway in tumor-combined immunotherapy; accordingly, increased studies focus on exploration of STING pathway agonists and analogues. However, current studies propose the potential use of the cGAS/STING pathway in tumor initiation and metastasis. Here, we review the molecular mechanisms and activation of the cGAS/STING pathway, and the relationship between DNA damage and this pathway, particularly highlighting the remodeling of immune contexture in tumor environment (TME) triggered by cascade inflammatory signals. A detailed understanding of TME reprogramming initiated by this pathway may pave the way for the development of new therapeutic strategies and rational clinical application.

## 1 Introduction

The tumor environment (TME) is known as a highly dynamic and constantly evolving system that is hard to predict. Interactions between various types of cells or cells with non-cells affect tumor growth and progression. In the process of tumor progression and oncotherapy, the DNA damage of tumor cells occurs frequently induced by various stresses; meanwhile, the immune system is activated continuously. DNA damage has been concluded as a critical factor in immune activation. Currently, inflammation response has become an important characteristic of tumor, and abnormal inflammatory mediator expression has been considered to be directly related to tumor prognosis (Qu et al., 2018; Greten and Grivennikov, 2019). The tumor could affect all systems in an organism, including the immune system, and when combined with radiotherapy or chemotherapy, it may lead to the collapse of the immune system. Experimental and clinical studies have suggested that a great part of deaths occurring in cancer are related to chronic infections, which are unmanageable and frequently in an advanced tumor stage. Indeed, interactional signals produced by tumor cells and immune cells in TME induce the changes of TME and build a tumor “preferred” TME to support growth and metastasis (Hinshaw and Shevde, 2019; Chen et al., 2021). Throughout the tumor process, the TME continues to evolve and reconstruct in the context of DNA damage, and the host struggles against the tumor persistently. Researchers have attempted to reveal the relationship among DNA damage, inflammation, and tumors, but it remains unclear.

The cGAS/STING pathway, a cytosolic DNA receptor, has been regarded as an important mechanism to regulate inflammation-driven tumor progression (Ahn et al., 2014). The cyclic GMP-AMP synthase (cGAS) is known due to its specific ability of recognizing and responding to cytosolic DNA in a DNA-sequence-independent but DNA-length-dependent manner (Sun et al., 2013). STING is an adaptor in innate immune which inherits the activation signal of cGAS and triggers downstream immune inflammatory response to protect the host. The function of the cGAS/STING pathway in eliciting immunity against exogenous pathogenic microorganisms has been extensively reported. Recent lines of evidence have extended the role of this pathway to cancer, senescence, and autophagy. In this review, we focus on the dichotomous roles of cGAS/STING in TME remodeling and its profound influence as a potential therapeutic strategy against cancer.

## 2 Overview of the cGAS/STING Pathway

cGAS, a 522-amino-acid protein, contains an unstructured positively charged domain (N-terminal) and a nucleotidyltransferase domain (C-terminal), both of which are working to bind with DNA. The N-terminal domain is reported to be involved in cGAS nuclear translocation (Gentili et al., 2019). The C-terminal domain contains two lobes with an active site as the catalytic domain of cGAS. The N-terminal domain contributes to the separation of the cGAS/DNA complex to mediate the cGAS activation once bound with DNA (Du and Chen, 2018). After binding with DNA, cGAS assembles into a dimer, which is formed by two DNA fragments embedded into two cGAS molecules to maintain a stable active state. It was reported that the longer DNA performed more efficiently in cGAS activation and promotion of cGAS/DNA complex formation (Zhou et al., 2018).

It has been concluded that cGAS is located in the cytoplasm and is kept isolated from self-DNA in the nucleus and mitochondria to prevent cGAS activation. However, recent studies presented that cGAS could be observed in the nucleus in case of DNA damage (Liu H et al., 2018; Zierhut et al., 2019). What is more, it was indicated that cGAS was mainly localized in the nucleus but strictly separated from chromatin (Volkman et al., 2019). However, the mechanisms through which cGAS could remain inactive in the nucleus remain unclear. It is speculated that the predominant localization of cGAS in the nucleus might be a preparation for rapid response to guarantee sufficient signaling under conditions of DNA exposure (Hopfner and Hornung, 2020).

After binding with DNA, the cGAS dimer catalyzes ATP and GTP into 2′,3′-cyclic GMP-AMP (cGAMP), a second messenger, to activate stimulator of interferon genes (STING) at the endoplasmic reticulum (ER) and initiate STING re-localization in the cytoplasm. STING is a 40-kDa protein with four transmembrane domains in ER, which are responsible for binding kinase TANK-binding kinase 1 (TBK1) (Zhang et al., 2020). Upon binding to cGAMP, STING is activated through transforming the structure from a higher-order oligomerization to tetramers (Shang et al., 2019; Zhao et al., 2019). Then, STING is transferred from ER to Golgi, where STING recruits and activates TBK1, and then promotes interferon regulatory factor 3 (IRF3) and NFκB translocation into the nucleus and conducts transcriptional function further (Li et al., 2013; Liu et al., 2015; Zhang et al., 2019).

## 3 Activation of the cGAS/STING Signaling Pathway

### 3.1 cGAS Recognizes DNA Fragment

cGAS/STING pathway response is concluded to be activated *via* DNA fragments. It is clear that the DNA source of pathogenic microorganisms is the primary factor of the pathway activation. Recent studies indicated that cGAS can also interact with endogenous self-DNA fragments, including nuclear DNA, mitochondrial DNA, micronucleus, and chromatin free in cytoplasm.

It has been confirmed that cGAS could combine with double-stranded DNA (dsDNA), single-stranded DNA (ssDNA), and RNA–DNA hybrids in the cytoplasm (Herzner et al., 2015; Luecke et al., 2017). Various exogenous DNA that could bind with cGAS were suggested, including bacteria, viruses, and parasites (Hahn et al., 2018; Cohen et al., 2019; Song et al., 2020). cGAS expression is also detected in the nucleus; it is assumed that exogenous DNA from viruses might be identified in the nucleus by cGAS, due to the increased accessibility as the virus replicates in the nucleus (Lahaye et al., 2018). The exogenous DNA released into intercellular space could also activate immune cells and neighboring cells to initiate the defense response of the host (Nandakumar et al., 2019).

**FIGURE 1:**
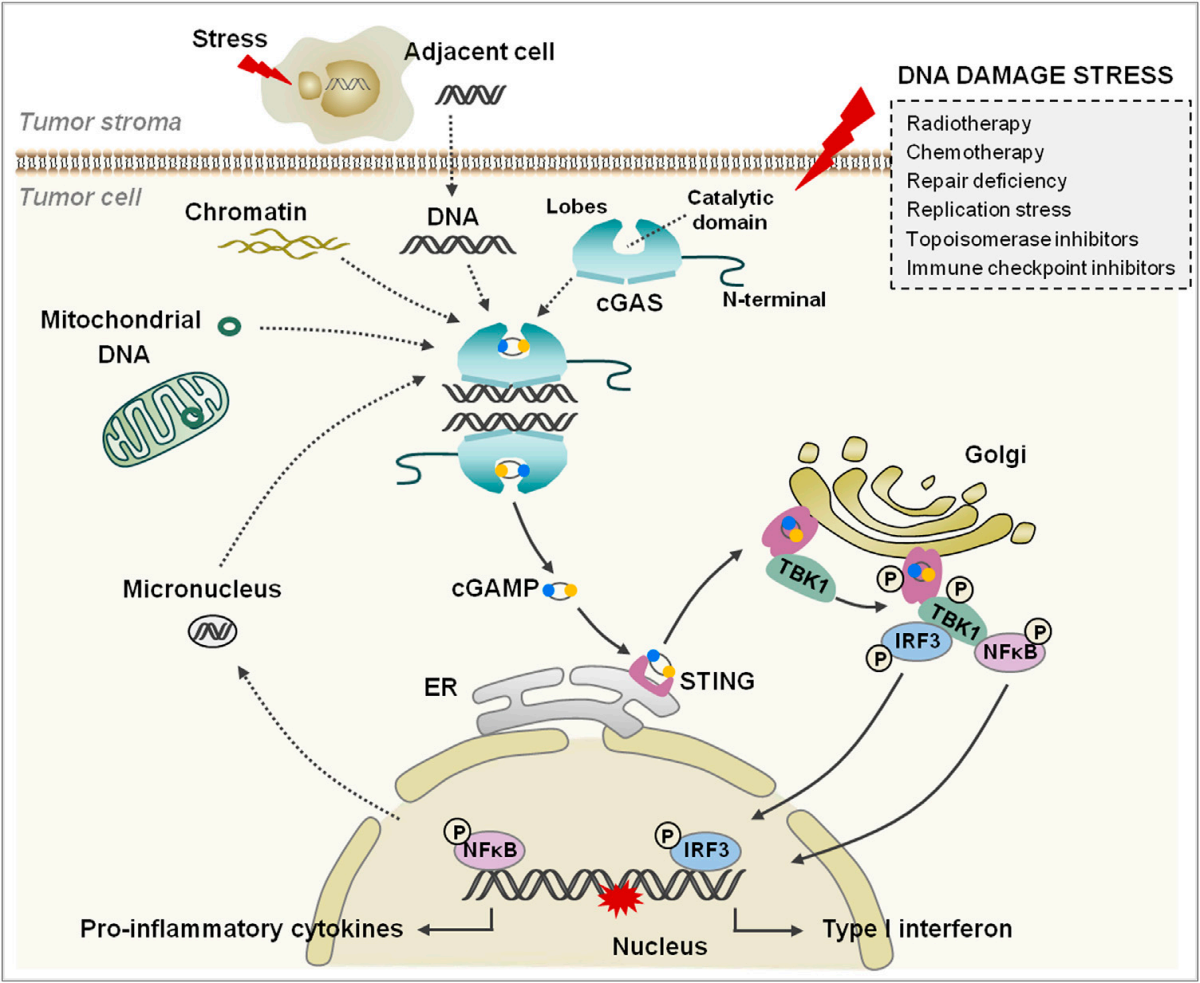
The cGAS/STING signaling pathway. cGAS consists of an N-terminal domain and a C-terminal domain that contains two lobes and a catalytic domain. The tumor cells are damaged under various stresses (the box on the right); the free self-DNA from the nucleus, mitochondria, and dying tumor cells bind to and activate cGAS, and catalyze the synthesis cGAMP. cGAMP binds to and changes the conformation of STING, and then STING transfers from ER to Golgi apparatus and is phosphorylated by adjacent activated TBK1. Subsequently, IRF3 and NFκB are phosphorylated by TBK1 and translocate into the nucleus to regulate IFN-I and inflammatory cytokine generation.

Recently, increasing lines of evidence indicate that endogenous self-DNA plays a crucial role in activating the cGAS/STING pathway, which is closely linked to health and disease. Self-DNA is commonly packaged or restricted in the nucleus and mitochondria to constrain the contact with cGAS (Boyer et al., 2020; Michalski et al., 2020). A recent study indicated that cGAS was not free in the cytoplasm but localized on the plasma membrane through the N-terminal domain (Barnett et al., 2019). If these restrictions are violated, thus triggering self-DNA or cGAS release into cytoplasm, judged as mislocation, a rapid and intense inflammatory reaction would be initiated *via* the cGAS/STING pathway (Zhang et al., 2019). Normally, the self-DNA mislocation could be induced by various stress factors, such as ultraviolet light, ionizing radiation, DNA damage agents, and replication stress; the subsequent DNA repair failure and cell death are the other important sources of free self-DNA (Bhattacharya et al., 2017; Mackenzie et al., 2017). In the process, increased genomic instability leads to exposure of chromatin and formation of abnormal micronucleus, which are also regarded as the agonist of the cGAS/STING pathway (Figure 1).

Another potential source of self-DNA in the cytoplasm is mitochondrial DNA (mtDNA) (Figure 1). Mitochondrial degeneration and membrane potential reduction is the primary cause of mtDNA leaking into the cytoplasm. Studies have performed that the opening of mitochondrial permeability transition pore (mPTP) could lead to mtDNA release; voltage-dependent anion channel 1 (VDAC1) oligomers were also involved in the process through formation of pores in the mitochondrial outer membrane (Kim J et al., 2019). Consistently, a recent study showed that the hyperinflammatory responses were induced in amyotrophic lateral sclerosis through cGAS/STING pathway activation *via* mPTP- and VDAC1-mediated mtDNA release (Yu et al., 2020). In the process, the dimer in the mitochondrial outer membrane was formed by bax and bak, which contribute to open the pores on the membrane and free mtDNA from the mitochondrial matrix (White et al., 2014; McArthur et al., 2018). Simultaneously, the mitochondrial cytochrome c is also leaked into the cytoplasm and activates caspases to cleave cGAS and IRF3 to block inflammatory reactions (White et al., 2014; McArthur et al., 2018).

### 3.2 Activation of the cGAS/STING Pathway

The C-terminal of cGAS contains a motif with zinc ion binding module, which is involved in DNA binding and cGAS dimerization. The pocket between two lobes is the pivotal binding site of substrates (Hopfner and Hornung, 2020). Once cGAS binds with DNA, the pocket structure of cGAS would transform to cyclize ATP and GTP into cGAMP (Figure 1). The cGAMP contains two phosphodiester bonds; one connects 2′-hydroxyl of GMP to 5′-phosphate of AMP, and another connects 3′-hydroxyl of AMP to 5′-phosphate of GMP(Ablasser et al., 2013a; Zhang et al., 2013). Therefore, this unique isomer determines the specific activation of cGAS by dsDNA, although the ssDNA could also bind with cGAS, but the lack of specific phosphodiester bonds makes the activation impossible under the circumstances (Zhang et al., 2020).

Recent studies reported that interaction of cGAS and dsDNA induced the formation of micrometer-sized liquid-like droplets through liquid–liquid phase separation, in which cGAS was activated (Du and Chen, 2018). These lipid-like droplets enhance cGAMP generation through increasing the concentrations of reactants, and the process is reported to be dynamic and reversible, which is proposed to initiate or terminate inflammatory response to DNA in a timely manner (Du and Chen, 2018).

The cGAMP binds with STING to form a polymer, in which the pocket conformation of STING would be changed from an open roof to a closed conformation (Shang et al., 2012; Zhang et al., 2013). Subsequently, STING leaves ER and transfers to the Golgi apparatus in the form of COP-II vesicles, where the STING dimer would be phosphorylated by the adjacent activated TBK1 but not the one bound itself (Liu et al., 2015). The phosphorylation of this complex provides a docking site for recruiting IRF3 *via* binding with the positively charged surface of IRF3, and then IRF3 is phosphorylated by TBK1; thus, the dimerized IRF3 translocates into the nucleus and turns on interferon-I (IFN-I) and inflammatory cytokines (Tao et al., 2016). Another alternative mechanism is to activate NFκB downstream of this pathway, but the contradictory models in the process have been previously proposed (Konno et al., 2013; Fang et al., 2017; de Oliveira Mann et al., 2019) (Figure 1).

## 4 DNA Damage and cGAS/STING

As the storage bank of genetic information, maintaining the integrity of DNA is of importance. Emerging lines of evidence have suggested that the cGAS/STING pathway plays a pivotal role in regulating DNA damage response and genomic instability, which is involved in the progression of multiple diseases including cancer.

### 4.1 DNA Damage Response and Genomic Instability

DNA damage of cells can be induced by exogenous and endogenous stress; cells establish a complex DNA damage response (DDR) system in the process, which involves multiple interactive or independent signaling pathways, and much of them remain unclear. Various cell biological processes are in connection with DDR, such as cell cycle regulation, DNA damage repair, cell metabolisms, senescence, and apoptosis. Timely and appropriate DDR has a positive effect on maintaining integrity and correctness of genome.

Genomic instability is an important indicator in disease events particularly in cancer, which has been observed in a variety of malignancies and precancerous lesions, and is related to prognosis, therapy, and overcome (Liu X et al., 2017; Kim J. H et al., 2019; Bao et al., 2021). Genomic instability elevation could be due to the defect of DDR and increased replication stress. Normally, the intracellular random errors produced by replication or stress exposure would trigger cell cycle checkpoints and DNA damage repair system to correct and rescue to ensure genetic stability. The abnormal damage response and repair could induce genomic instability occurrence through breaking the limited fidelity of DNA. It is realized that most of the human tumors are associated with genomic instabilities, which also indicate the tumor stage, metastasis, and recurrence (Chan-Seng-Yue et al., 2020; Bao et al., 2021). Genomic instability is related to the resistance of chemotherapy and radiotherapy in a clinical setting, such as taxol, 5-fluorouracil, and epirubicin used in breast cancer, colon cancer, and osteosarcoma (Telli et al., 2016; Hoglander et al., 2018). The increased genomic instability, abnormal chromosome copy numbers, and chromosome deficiency have also been verified in some metastasis of tumors (Pailler et al., 2015; Bakhoum et al., 2018).

As another result of DNA damage, small fragments of DNA leak out of the nucleus in mitosis and form the membrane-packaged micronuclei (Hintzsche et al., 2017). As mentioned previously, micronucleus is a pivotal source of self-DNA, through which cGAS is activated and triggers downstream signaling pathway to initiate inflammatory immune response. cGAS is confirmed to be co-localized with γH2AX, a DNA damage marker. Furthermore, researchers showed that the co-localization of cGAS with γH2AX did not exist only in micronuclei in the cytoplasm; it was also observed that cGAS was transferred into the nucleus and localized at the sites of damaged dsDNA (Liu H et al., 2018). The DDR to micronuclei that connected with the cGAS/STING pathway might guide the fate selected by cells to deal with, rescue or elimination; consequently, the irreparable DNA damage of cells leads to apoptosis but failed rescue induces mutation and tumor eventually (Gulen et al., 2017).

### 4.2 Interaction of the cGAS/STING Pathway and Tumor

Increased number of studies reveal the crucial role of the cGAS/STING pathway in innate antitumor immunity; however, evidence on the cGAS/STING pathway promoting tumor progression is also emerging.

The DNA of tumor cells is commonly released in the process of rapid proliferation and antitumor therapy; subsequently, cGAS recognizes the DNA source and responds quickly to activate STING and downstream cascade reaction to eliminate tumor cells through innate immune response (Wang et al., 2020). Researchers have shown that micronuclei are widespread in tumor cells and tumor stroma. Antigen-presenting cells (APC) are initiated and regulated by IFN-I, and then the tumor antigens yield to CD8 T cells and natural killer (NK) cells (Woo et al., 2014; Marcus et al., 2018). Recent studies have performed that the cGAS/STING pathway is activated in APC *via* free DNA in tumor, which renders tumor vulnerable to immunological surveillance (Marcus et al., 2018). cGAS/STING pathway activation in tumor cells forms an obstacle to the early-stage tumors through upregulating IFN-I and inflammatory cytokines for antitumor immunity, which is also closely related to induction of tumor cell senescence (Dou et al., 2017).

On the other hand, the tumor cells need to evade this signaling pathway detection to survive in the harsh living environment; thus, IFN-I deletion and the cGAS/STING axis are observed to be disrupted in tumors (Gajewski and Corrales, 2015). Previous studies showed that the cGAS/STING pathway could be rendered defectively by various mechanisms, such as the interrupted translocation from ER to Golgi, abnormal methylation at promoter regions of cGAS and STING, and improper posttranslational modification of these proteins (Xia et al., 2016a; Xia et al., 2016b). A recent study suggested that hypoxia in TME could inactivate the cGAS/STING pathway and induce immunosuppression through targeting an epigenetic factor NCOA3 by hypoxia-responsive miRNAs, which was necessary for basal levels of cGAS expression (Wu et al., 2017). As expected, restoring cGAS expression recovered the anti-tumor immune response (Wu et al., 2017). In addition, cGAS/STING pathway activation has also been indicated to regulate intrinsic cellular programs, including inducing tumor cell autophagy, apoptosis, necroptosis, and pyroptosis (Vanpouille-Box et al., 2018; Li et al., 2019; Zhang et al., 2020).

It seems certain that the success of radiotherapy and chemotherapy in tumor therapy is closely related to the innate immune signaling partially mediated by the cGAS/STING pathway. Meanwhile, evidence that the cGAS/STING pathway-mediated immune inflammation contributed to tumorigenesis, progression, and metastasis in some tumors was proposed; thus, the application involved in this pathway in oncotherapy became more complicated (see below). In general, the tumor immunotherapeutics need to achieve a rational balance between promoting potent antitumor response and preventing inflammation-mediated tumor progression.

## 5 Remodeling of TME Induced by DNA Damage Through the cGAS/STING Pathway

### 5.1 Alternation of Metabolites in TME

The TME is as a nutrient-rich soil affording nutrition to tumor cell growth after reconstruction by tumor, in which the antitumor immunity is restrained. Proteins and amino acids are crucial for tumor proliferation and reconstruction of TME, which could remodel tumor stroma and angiopoiesis as the tumor develops, to construct a proper environment for its growth (Bose et al., 2020; Hou et al., 2020; Winkler et al., 2020). The catabolism of amino acid tryptophan (Trp) is a common feature in antitumor immunity defeat (Garber, 2018; Mitchell et al., 2018). Trp can be catabolized by indoleamine 2,3 dioxygenase (IDO) enzyme produced from tumor cells; the metabolic kynurenine has been confirmed to suppress T-cell proliferation and function (Liu Y et al., 2018; Mitchell et al., 2018; Takenaka et al., 2019). Arginine (Arg) is catabolized into L-ornithine by arginase (ARG1) as well as nitric oxide synthetase (NOS), which performs immunoregulation of M2 macrophages and myeloid-derived suppressor cells (MDSCs); L-ornithine could mediate the tumor cell proliferation and suppression of antitumor immunity *via* converting into polyamines (Roci et al., 2019; Fultang et al., 2020; Carriche et al., 2021; Miska et al., 2021).

The increased cell death in TME induces the DNA release and cGAS/STING pathway activation to initiate innate immunity subsequently. The innate immune cells could produce IFN-I (IFN-α and IFN-β) and IFN-II (IFN-γ), which stimulate downstream gene production including gene encoding enzymes to catabolize Trp and Arg, inflammatory cytokines, and transforming growth factor-β (TGF-β) (Weiner, 2009). Both IFN-α and IFN-γ could induce IDO1 expression, but IDO1 is restrained by the regulation factors of IFN-β (Liu Y et al., 2017; Du et al., 2019; Shi et al., 2019; Cheng et al., 2020). In addition, the Trp catabolism is increased in cancer patients, as a precursor of 5-hydroxytryptamine, and its overexpression could lead to emotion changes and depressive behaviors in patients (Tang et al., 2020; Karmakar and Lal, 2021). IFN-γ promotes the inducible NOS (iNOS) expression, while cytokines IL-4 and IL-13 stimulate ARG1; moreover, TGF-β enhances both IDO1 and ARG1 response (Boutard et al., 1995; Ji et al., 2019; Baier et al., 2020). The current studies have indicated that these pathways are all involved in remodeling the expression of metabolites in TME that is activated *via* tumor-associated inflammation, and interfere with tumor therapy and prognosis; however, whether more metabolites play a synergy role in this process remains to be clarified.

### 5.2 Implication to Immune Cells in TME

#### 5.2.1 Antigen-Presenting Cell (APC)

APCs play a critical role in the uptake and processing of antigens and then present to T cells for immune response. Generally, the damaged and dying non-tumorigenic cells could avoid activation of APCs to prevent the autoinflammatory disease because of chronic cytokine production. It has been confirmed that antigen presentation on the surface of tumor cells could be enhanced in radiotherapy and chemotherapy, and then T cells recognize antigen presented on major histocompatibility complex I (MHC-I) and respond rapidly.

**FIGURE 2:**
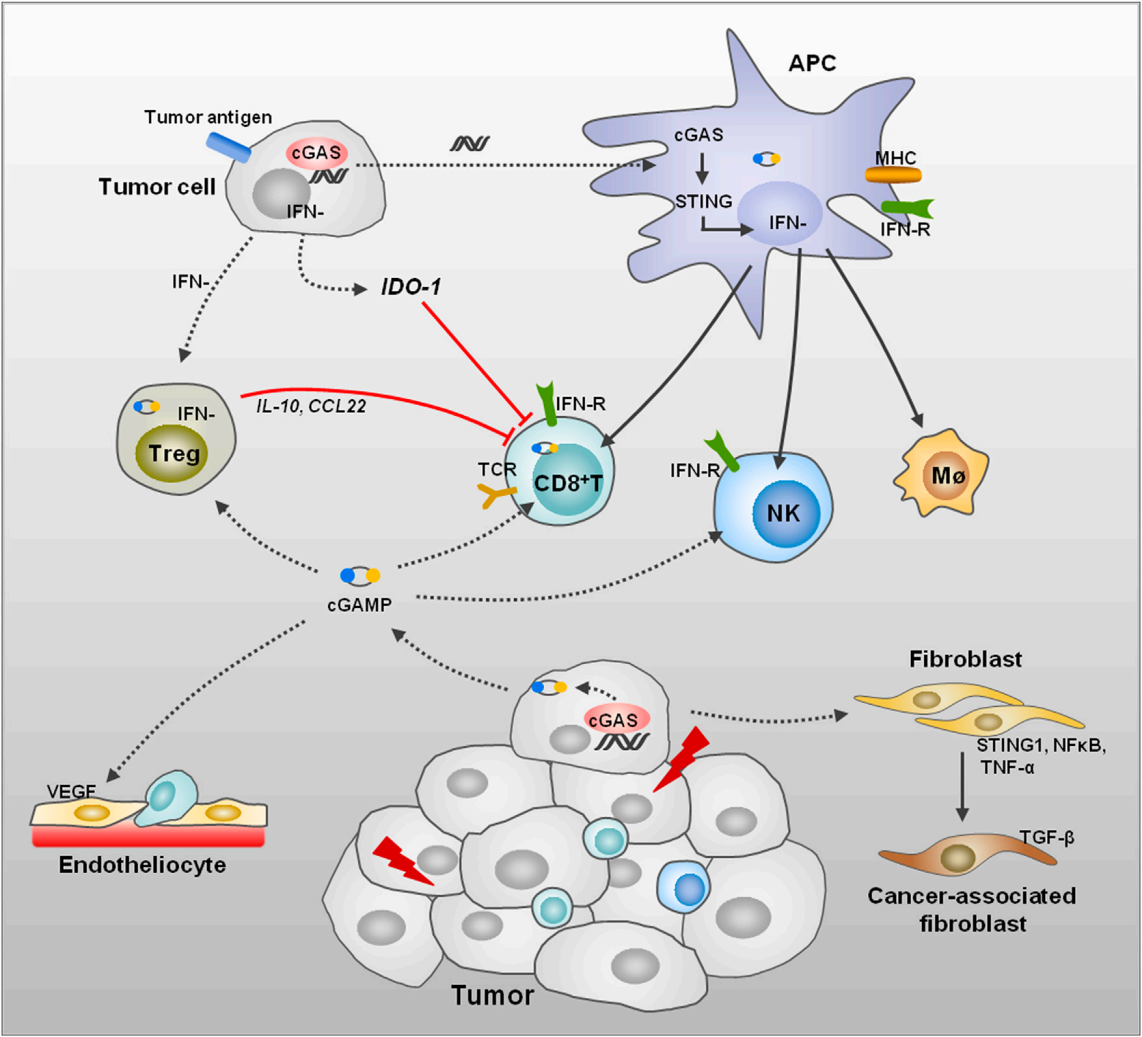
Remodeling of TME induced by DNA damage. DNA damage of tumor cells leads to dsDNA, thus activating the cGAS/STING signaling pathway and promotes IFN generation in several kinds of cells. The APC activation can be induced through endocytosis of tumor-derived dsDNA, cGAMP, or extracellular vesicle. Then, APCs initiate CD8^+^ T cells, NK cells, and macrophages to enhance the immune response in TME. The Treg cell activation induced by tumor cells performs immune suppression to T-cell proliferation and functions through anti-inflammatory factors. The tumor cells also induce IDO1 expression to enhance amino acid metabolism, thus suppressing T-cell function. STING activation promotes normalization of tumor vasculature and increases migration of T cells across endothelial barrier and enhances antitumor immunity. In addition, cGAS/STING pathway activation in fibroblasts affects the differentiation of fibroblasts to CAFs.

Recent studies suggested that multiple oncotherapies were related to activation of the cGAS/STING pathway, as the tumor-derived DNA was detected in the cytoplasm of the tumor-infiltrating dendritic cells (DCs); meanwhile, tumor-specific antigen presentation and cytotoxic T-cell activation were increased (Chen et al., 2016b; Deng et al., 2014; Wang et al., 2017) (Figure 2). In chemotherapy of ovarian cancer, cisplatin exposure boosted tumor immunogenicity *via* elevating calreticulin, MHC-I, antigen presentation, and T-cell infiltration through activating the cGAS/STING pathway (Grabosch et al., 2019). In the process of photodynamic therapy (PDT), PDT enhanced MHC-II and CD80 expression and induced maturation of DCs in an IFN-I-dependent manner in melanoma (Lamberti et al., 2019). In TME, mtDNA of tumor cells were ingested by DCs and activate cGAS to increase IFN-I production in DC cytoplasm; inhibition of CD47 could suppress mtDNA degradation by phagosomes, which contributes to enhance antitumor adaptive immunity (Xu et al., 2017).

The IFN-I plays an important role in activating innate and adaptive immune through promoting maturation and activation of DCs and macrophages, thus enhancing the antigen presentation and T-cell infiltration (Figure 2). Manganese (Mn^2+^) is a potent activator of cGAS, which could be released from mitochondria and Golgi and bind with cGAS in the cytoplasm to enhance enzymatic activity of cGAS (Wang et al., 2018). Mn^2+^ treatment stimulates IFN-I and cytokine production *via* the cGAS/STING pathway and improves response to clinical immunotherapy in patients (Lv et al., 2020). In a recent study, *Bacillus* Calmette-Guérin (BCG) instillations in urothelial carcinoma elevated STING and IFN as well as pro-inflammatory molecules, thus promoting M1 macrophages and T-cell infiltration in tumor (Lombardo et al., 2021). In addition, in non-muscle invasive bladder cancer, expression of STING was higher in patients who responded to BCG therapy, and elevated further after BCG treatment (Lombardo et al., 2021).

Studies have performed that the STING agonist (2′3′-cGAMP) could facilitate malignant B-cell apoptosis by phosphorylation and activation of STING on mice fibroblasts; subsequently, the tumor cell antigens are released to stimulate immune response in this manner (Tang et al., 2016). A recent study proposed that treatment with STING agonist decreased tumor burden in high-grade serous carcinoma, and mice were able to survive *via* the combination treatment of carboplatin, STING agonist, and anti-PD-1. In the process, STING agonist treatment enhanced IFN response, antigen presentation, and MHC II expression (Ghaffari et al., 2018).

#### 5.2.2 T Cell

In solid tumor therapy, T-cell-based immunotherapy made a breakthrough but encountered multiple challenges; the specific targetable tumor antigen presentation is of importance to T-cell therapy (Lim and June, 2017; Sadelain et al., 2017). Current studies indicate that spontaneous initiation of tumor antigen-specific T cells is likely to be relevant to DC antigen presentation and IFN-I production in host cells (Diamond et al., 2011).

A recent research indicated that the cGAS/STING cascade was remarkably suppressed in peripheral blood CD8^+^ T cells from tumor patients, STING agonist treatment promoted CD8^+^ T cell stemness from patients with cancer; in addition, elevated STING activation enhanced oncotherapy of CAR-T cells in a xenograft model (Li W et al., 2020). In triple-negative breast cancer therapy, the PARP inhibitor olaparib induced T-cell infiltration *via* the cGAS/STING pathway in tumor and paracrine activation of DCs was enhanced in the process; furthermore, activation of the pathway was more obvious in homologous recombination-deficient tumor cells (Pantelidou et al., 2019). Consistently, pro-inflammatory response and T-cell recruitment were abolished after knockout of STING in tumor cells (Pantelidou et al., 2019). In another study, CD8^+^ T-cell infiltration in engrafted melanoma was lower than that in wild-type mice, but intratumoral injection of cGAMP facilitated immune response. Mechanistically, the cGAS/STING pathway was activated by STING agonist in endothelial cells instead of DCs and other immune cells, thus promoting the trafficking and infiltration of CD8^+^ T cells into tumor (Demaria et al., 2015).

Based on the reported assay of immunogenic cell death and T-cell activation, a DNA topoisomerase II inhibitor was proposed to induce the protein HMGB1 release and IFN-I expression in tumor; subsequently, DCs were activated through both NFκB activation and the STING-dependent IFN-I pathway, and then T cells were recruited into the tumor to increase therapeutic efficacy (Wang et al., 2019). Ataxia telangiectasia mutated (ATM) is known as a critical factor in nucleus DNA damage repair; surprisingly, blockade of ATM is indicated to facilitate immune checkpoint blockade therapy. Mechanistically, inhibition of ATM promotes mtDNA leakage into the cytoplasm and activates the cGAS/STING pathway *via* suppressing mitochondrial transcription factor A (TFAM), thus enhancing T-cell infiltration into TME subsequently (Hu et al., 2021). Another critical kinase in DDR is ATR; the ATR inhibitor performs radiosensitization to tumor alongside remarkable infiltration of CD3^+^ and NK cells in TME through activation of STING and inducing IFN response (Dillon et al., 2019). Furthermore, inhibition of RAD51, a critical component in DNA double-strand break repair, activated the cGAS sensing pathway and improved CD8^+^ T-cell infiltration *via* increasing cytosolic dsDNA in small cell lung cancer (Jin et al., 2021). Majority of studies display the positive function of the cGAS/STING pathway in facilitating T-cell activation and recruitment in TME; undoubtedly, the negative regulation of this pathway to T cells is also presented (Figure 2) (see below).

#### 5.2.3 Regulatory T Cell (Treg Cell)

Treg cells suppress immune reaction; generally, the ratio of Treg and T cells keeps a dynamic change to maintain immune response stability in the body. A previous study indicated that Treg cells can activate and facilitate proliferation by tumor-associated antigens in TME, which leads to immune tolerance and treatment resistance of tumors (Ahmadzadeh et al., 2019) (Figure 2). Combination therapy including STING agonist, anti-PD-1, and anti-CTLA-4 led to significant tumor regression in mice; the Treg cell ratio was suppressed obviously with increased CD8^+^ T cells in oropharyngeal cancers (Dorta-Estremera et al., 2019). In a glioma study, using the tdTomato mice, it was indicated that the IFN-I signal triggered by STING blocked Treg cells and promoted CD8^+^ T-cell response; furthermore, the efficacy of OVA-targeted peptide vaccine was enhanced by STING agonist (Ohkuri et al., 2014). A different opinion presented that IFN-β transcript sustained in resistant tumors induced PD-L1 and NOS2 expression in tumors and DCs that affected Treg cell accumulation in TME, thus enhancing the ratio of CD8^+^T/Treg cells in the context of long-term anti-PD-1 treatment (Jacquelot et al., 2019).

### 5.3 Angiogenesis

In TME, tumor growth is dependent on angiogenesis and competitive nutrition, and the chronic immune response induces growth factors and results in angiogenesis and suppression of antitumor. Multiple proangiogenic factors in TME are involved in tumor angiogenesis to drive new blood vessel formation (Jiang et al., 2020; Ronca et al., 2017). Tumor blood vessels appear disorganized and immature, which reduces chemotaxis of immune cells into TME but increases the distant metastasis of tumor cells. A recent study proposed that T-cell transendothelial migration was regulated by endothelial STING in an IFN-I-dependent manner (Anastasiou et al., 2021). IFN-β was proposed to downregulate VEGF expression and suppress tumor angiogenesis (Takano et al., 2014), but it was also shown that IFN-α and IFN-β promoted vasculogenic mimicry formation and facilitated tumor growth (Jablonska et al., 2010; Yeh et al., 2018). Interestingly, STING activation plays a positive role, including promoting normalization of tumor vasculature and improving immune response in TME (Figure 2). Restoration of vascular structure results in increased migration of T cells across the endothelial barrier and enhances antitumor immunity (Yang et al., 2019). A genome-wide phenotype screen showed that TBK1, IRF3, and downstream signals were suggested to be the necessary proangiogenic factors (Korherr et al., 2006). However, another study showed that the activation of the cGAS/STING/IRF3 pathway induced by palmitic acid treatment suppressed angiogenesis mechanistically and activated IRF3 bound to the promoter of mammalian Ste20-like kinases 1 (MST1) gene, thus inhibiting endothelial cell proliferation (Yuan et al., 2017).

### 5.4 Reprogramming of Fibroblast in TME

Fibroblasts are of importance to maintain integrity in normal tissues, whereas, in inflammatory response, fibrotic disease and tumors are reprogrammed for different functions (Driskell and Watt, 2015; Sahai et al., 2020). The metabolites and proteins derived from tumor cells are indicated to alter the biological characteristics of fibroblasts by remodeling their metabolism and phenotype; in addition, studies have provided more evidence of the key metabolic connection between tumor cells and cancer-associated fibroblasts (CAFs) (Bertero et al., 2019; Li F et al., 2020; Zhang et al., 2021). TBK1, downstream of the cGAS/STING pathway, was recently reported as a potential regulator of fibroblast activation; inhibition of TBK1 activity reduced α-SMA stress fiber level and mitigated deposition of collagen and fibronectin in fibroblasts (Aravamudhan et al., 2020). A recent study that combined mass cytometry and single-cell mRNA sequencing analysis proposed that expression of CD105 was the distinctive indication in two diverse functional fibroblasts in both healthy tissues and tumors (Hutton et al., 2021). Interestingly, results showed that TGF-β signaling was enriched in CD105 positive cancer-associated fibroblasts (CAFs), which were permissive for tumor growth (Figure 2). However, in CD105-negative CAFs, the STING1, NFκB, IL-6, TNF-α, JAK2, and LTBR signals observed high expression and remarkably performed tumor suppression (Hutton et al., 2021). In another important research, IFN-β1 was specifically upregulated in CAFs that contacted tumor cells through STING/IRF3 pathway activation due to the transcytosis of tumor cell cytoplasm into CAFs. Intriguingly, this reprogramming did not occur in CAFs that have no contact with tumor cells, which resulted in two different CAFs phenotypes and functions that coexisted in TME (Arwert et al., 2020).

## 6 Emerging Pro-tumor Role of the cGAS/STING Pathway

The emerging lines of evidence show that the cGAS/STING pathway performs positive facilitation on immune response in tumors; nevertheless, current studies propose a potential promotion of this pathway in tumor initiation, progression, and metastasis (Chen et al., 2016a; Lemos et al., 2016).

Chronic and aberrant inflammation is closely related to tumorigenesis and development. In inflammatory colitis associated tumor model, deficiency of STING increased the susceptibility to tumorigenesis (Ahn et al., 2015), but in a non-inflammatory Lewis lung carcinoma (LLC), STING activation induced tumor growth (Lemos et al., 2016). It has been indicated that activated the cGAS/STING pathway accelerates initiation and activation of DCs and T cells, and recent studies showed that STING activation suppressed proliferation of T cells, which was independent with the TBK1/IRF3/IFN-I axis downstream, but in a manner of NFκB activation by the distinct C-terminal domain of STING in T cells (Cerboni et al., 2017). The STING agonist treatment induced initiation of IFN-I and T-cell-specific response involved in ER stress and cell death pathways, but only STING activation without cell antigen receptor would induce T-cell death in the process (Larkin et al., 2017). Researchers evaluated the relationship of STING expression and immune cell infiltration in malignant tumor, and suggested that pan-cancer expression of STING was positively correlated with immune cell infiltration including all types of immune cells (An et al., 2019). Inhibition of cGAS or STING expression in tumor cells could prevent metastasis in animal models (Chen et al., 2016a; Bakhoum et al., 2018).

The tumor metabolite in TME, such as the amino acids tryptophan and arginine, the common TME hallmarks in clinical oncotherapy, are proposed to respond to IFN and transforming growth factor-β (TGF-β) cytokines to suppress antitumor immunity and promote tumorigenesis (Rodriguez et al., 2007; Weiner, 2009; Opitz et al., 2011). An oral cancer study displayed that the oxidized mtDNA in cytosol induced IFN signaling through the cGAS/STING pathway and thus elevated PD-L1 and IDO-1 expression, which inhibited T-cell function through inducing IFN and IL-6 production from macrophages (Cheng et al., 2020). Another study indicated that STING activation did not impact cell viability in tongue squamous cell carcinoma, but facilitated IL-10, IDO, and CCL22 production, the immunosuppressive cytokines, thus inducing Treg cell infiltration and suppressing T-cell proliferation and activation (Liang et al., 2015). As previously mentioned, IDO plays a negative regulatory role in inflammatory response and T-cell activation. In mouse (LCC) models with STING knockout, suppressed IDO expression and MDSCs were observed, because IFN contributed to IDO induction. Furthermore, inhibition of IDO expression restrained tumor growth effectively, indicating the crucial role of IDO in TING-mediated tumor growth (Lemos et al., 2016). Therefore, IDO- and metabolite-induced immunosuppression in TME is an essential condition in the cGAS/STING pathway-involved tumorigenesis (Lemos et al., 2016).

A previous study indicated that tumor metastasis in mice brain was connected with the cGAMP transfer from tumor cells to astrocytes in an adjacent paracrine and endocytosis manner; in the process, the cGAS/STING pathway in astrocytes was activated as well as IFN-α and TNF-α, which contributed to establish a tumor growth advantage (Chen et al., 2016a). In addition, the activation of the STING/IFN-I pathway was also indicated to elevate CCR2 expression, and suppressive inflammation in colon tumors through recruiting MDSCs, CCR2 blockage-mitigated MDSC infiltration, and immunosuppression initiated by STING activation enhanced oncotherapy (Liang et al., 2017). Collectively, the potential immunosuppression of STING is emerging and is drawing more attention; in addition, the tumor cells surviving in antitumor therapy might change their tolerance and benefit from TME, which could facilitate tumor recurrence and metastasis. In this regard, sustaining dominance of the immunogenic process while minimizing the pro-tumor inflammation is of importance to oncotherapy.

## 7 The cGAS/STING Pathway in Oncotherapy

In the process of growth, progression, and therapy, the tumor cells would undergo various stresses and induce immune response to be removed in host. Recent studies propose that the cGAS/STING pathway plays crucial roles in antitumor immune response and immune surveillance. In TME, the tumor-derived DNA have been observed in APCs’ cytoplasm, and immune response is amplified though antigen presentation-induced recruitment of T cells and NK cells (Woo et al., 2014; Corrales et al., 2016). In addition, cGAMP was reported to be transmitted from cell to cell or to the extracellular area by some transport-associated and gap junction proteins, such as SLC19A1, CX43/CX45, LRRC8, and MerTK (Ablasser et al., 2013b; Chen et al., 2016a; Luteijn et al., 2019; Zhou C et al., 2020; Zhou Y et al., 2020).

The intensity of inflammation and the extent of cGAS/STING activation should be the critical factors in determining whether this pathway is antitumor or pro-tumor. Moreover, the genomic instability of tumor cells is another considerable element in cGAS/STING pathway-related pro-tumor and metastasis. In tumor progression, some tumor cells evolve to escape the host immune surveillance gradually; for example, the cGAS or STING expression is silenced or neglected so that the signal transduction cascade is interrupted and failed to trigger immune response (Xia et al., 2016a). Furthermore, DNA methylation has been proposed as a crucial factor to regulate the silencing of genes in tumor cells (Lai et al., 2021). As the crucial cytosolic DNA sensor of tumors, the ability to activate innate and adaptive immune responses of the cGAS/STING pathway attracts much attention for pharmacological target development. Currently, studies have mainly focused on the agonists of the cGAS/STING pathway and their usage as vaccine adjuvants for antitumor combined immunotherapy. The effect of tumor immunotherapy depends on expression of tumor-associated antigens and partially on antigen presentation; the cGAS/STING pathway has been used in combination immunotherapy in some tumors due to its enhancement of APC function.

Co-delivery of c-di-GMP and chimeric antigen receptor T (CAR-T) cells led to remarkably pancreatic tumor regression in mice (Smith et al., 2017). Combination of anti-PD-L1 and intramuscular injection of exogenous 2′3′-cGAMP suppressed melanoma growth and increased survival of mice harboring tumors (Wang et al., 2017). In pre-clinical models of ovarian tumor and aggressive lung cancer, combination therapy including anti-IL-10, 2′3′-cGAMP, and anti-PD-L1 targeting innate and adaptive immunity dramatically decreased MDSCs and improved DC activation and T-cell infiltration (Hartl et al., 2019). Breast tumor patients with high expression of CD47 showed poor survival and prognosis; cGAMP and anti-CD47 combination therapy effectively suppressed tumor growth, whereas monotherapy with anti-CD47 did not inhibit tumors (Kosaka et al., 2021). The flavone-8-acetic acid derivative 5,6-dimethylxanthenone-4-acetic acid (DMXAA), a selective STING agonist of mice, has outstanding antitumor characteristics in multiple tumor models (Curran et al., 2016; Weiss et al., 2017; Liu et al., 2020; Xu et al., 2021). In addition, ADU-S100, one of promising agonists of STING, exhibits significant inhibition on colon tumor and ascites in the case of synergistically cooperating with anti-PD-1 and anti-COX2 (Lee et al., 2021), which is investigated in clinical phase I trials of solid tumors and lymphomas (Sivick et al., 2018; Meric-Bernstam et al., 2021).

Over the past decade, more efforts are focused on the development of STING agonists that perform improved stability and binding capacity on human STING, some of which have been used in clinical trials of oncotherapy (Table 1). Correctively, antitumor immune therapy requires activating APCs by the cGAS/STING pathway as well as enhancing tumor-associated antigen presentation to T cells to improve efficiency.

**TABLE 1:** Clinical trials testing STING agonists in oncotherapy.

Agonists	Co-therapy	Tumor types	Phase	NCT Number
DMXAA	+Docetaxel	Advanced solid tumors	I	NCT01285453
	+Carboplatin + paclitaxel or docetaxel	Advanced solid tumors	I	NCT01240642
	+Carboplatin and paclitaxel	HNSCC	I	NCT00674102
	+Carboplatin and paclitaxel	HNSCC	I/II	NCT00832494
	+Carboplatin and paclitaxel	HNSCC	III	NCT00662597
	+Docetaxel	Prostate cancer	II	NCT00111618
	+Carboplatin and paclitaxel	SCLC	II	NCT01057342
ADU-S100	+Ipilimumab	Advanced solid tumors	I	NCT02675439
	+Spartalizumab	Advanced solid tumors or lymphoma	Ib	NCT03172936
	+Pembrolizumab	HNSCC	II	NCT03937141
MK-1454	+Pembrolizumab	Advanced solid tumors or lymphoma	I	NCT03010176
	+Pembrolizumab	HNSCC	II	NCT04220866
MK-2118	+Pembrolizumab	Advanced solid tumors or lymphoma	I	NCT03249792
SB11285	+Atezolizumab	Advanced solid tumors	Ia/Ib	NCT04096638
GSK3745417	+Pembrolizumab	Advanced solid tumors	I	NCT03843359
BMS-986301	+Nivolumab/ipilimumab	Advanced solid tumors	I	NCT03956680
E7766	Single agent	Advanced solid tumors, lymphomas, bladder cancer	I/Ib	NCT04144140

Abbreviations: HNSCC, head and neck squamous cell carcinoma, SCLC, small cell lung carcinoma.

## 8 Conclusion and Future Perspective

The DNA damage repair responses of cells have profound influence on inflammatory response and tumorigenesis. Defective DDR allows genomic instability and micronuclei formation, the pivotal source of self-DNA, through which the DNA sensor cGAS is activated and triggering downstream signal cascade reaction; what is more, the variability of TME exacerbates DNA damage and genomic instability. Accumulating studies have elucidated the crucial role of the cGAS/STING pathway in surveillance of free self-DNA. Emerging lines of evidence have indicated that activation of the cGAS/STING pathway facilitates antitumor immune responses effectively, except for the established role in innate immunity under condition of exogenous pathogens. Rapid progress has been acquired for understanding the molecular basis and mechanisms in antitumor immune responses, which provide novel insight and references to guide oncotherapy.

Notably, the chronic activation of inflammatory *via* the cGAS/STING pathway is closely related to tumorigenesis and metastasis. Moreover, the intensity of inflammatory reaction and cGAS/STING pathway activation in different cells lead to the exact opposite results, whereby the TME is remodeled in the process. The challenges promoting immunostimulatory effects of oncotherapy while blocking negative immunosuppression remain insurmountable. Hence, how to grasp the internal relationship among various objects in TME and balance the activation status of the pathway in cells with antithetical functions still needs in-depth investigation.

The discovery and investigation of the cGAS/STING pathway in tumor therapy and TME provide a novel framework for future therapeutic strategies. The inspiring potential of this pathway activation promotes intense investigation for the development of pharmacological compounds in this pathway. The agonists and analogues have been used as immune adjuvants in combined therapy such as chemotherapy, radiotherapy, and immune checkpoint blockade in preclinical trials to enhance efficacy. At present, the cGAS/STING pathway is considered to be a promising therapeutic target that might turn the immunologically “cold” tumor to a “hot” one. Although effective drugs have been used in trials, potential problems might hinder their application in the future. For example, the chemical property restrains the penetrating capacity, delivery mode, and bioavailability of drugs, including charged property, hydrophilicity, and metabolism. In addition, the cytotoxicity and narrow therapeutic windows restrict the application scope of drugs. Therefore, strategies to develop and screen potential agonists and to improve drug delivery carriers are urgently needed. On the other hand, emerging preclinical and clinical lines of evidence reveal that various antitumor drugs could activate this pathway through DNA damage; neglect of this potential may underestimate its contribution to therapeutic efficacy. Therefore, the combinatorial treatment for therapeutic benefit is considerable and promising.

## References

[B1] Ablasser A. Goldeck M. Cavlar T. Deimling T. Witte G. Röhl I. (2013a). cGAS Produces a 2′-5′-linked Cyclic Dinucleotide Second Messenger that Activates STING. Nature 498 (7454), 380–384. 10.1038/nature12306 23722158 PMC4143541

[B2] Ablasser A. Schmid-Burgk J. L. Hemmerling I. Horvath G. L. Schmidt T. Latz E. (2013b). Cell Intrinsic Immunity Spreads to Bystander Cells via the Intercellular Transfer of cGAMP. Nature 503 (7477), 530–534. 10.1038/nature12640 24077100 PMC4142317

[B3] Ahmadzadeh M. Pasetto A. Jia L. Deniger D. C. Stevanović S. Robbins P. F. (2019). Tumor-infiltrating Human CD4 + Regulatory T Cells Display a Distinct TCR Repertoire and Exhibit Tumor and Neoantigen Reactivity. Sci. Immunol. 4 (31). 10.1126/sciimmunol.aao4310 PMC6685542 30635355

[B4] Ahn J. Xia T. Konno H. Konno K. Ruiz P. Barber G. N. (2014). Inflammation-driven Carcinogenesis Is Mediated through STING. Nat. Commun. 5, 5166. 10.1038/ncomms6166 25300616 PMC4998973

[B5] Ahn J. Konno H. Barber G. N. (2015). Diverse Roles of STING-dependent Signaling on the Development of Cancer. Oncogene 34 (41), 5302–5308. 10.1038/onc.2014.457 25639870 PMC4998969

[B6] An X. Zhu Y. Zheng T. Wang G. Zhang M. Li J. (2019). An Analysis of the Expression and Association with Immune Cell Infiltration of the cGAS/STING Pathway in Pan-Cancer. Mol. Ther. - Nucleic Acids 14, 80–89. 10.1016/j.omtn.2018.11.003 30583098 PMC6305687

[B7] Anastasiou M. Newton G. A. Kaur K. Carrillo-Salinas F. J. Smolgovsky S. A. Bayer A. L. (2021). Endothelial STING Controls Tcell Transmigration in an IFN-I Dependent Manner. JCI insight 6 (15). 10.1172/jci.insight.149346 PMC8410041 34156982

[B8] Aravamudhan A. Haak A. J. Choi K. M. Meridew J. A. Caporarello N. Jones D. L. (2020). TBK1 Regulates YAP/TAZ and Fibrogenic Fibroblast Activation. Am. J. Physiology-Lung Cell Mol. Physiol. 318 (5), L852–L863. 10.1152/ajplung.00324.2019 PMC7272740 32159970

[B9] Arwert E. N. Milford E. L. Rullan A. Derzsi S. Hooper S. Kato T. (2020). STING and IRF3 in Stromal Fibroblasts Enable Sensing of Genomic Stress in Cancer Cells to Undermine Oncolytic Viral Therapy. Nat. Cel Biol 22 (7), 758–766. 10.1038/s41556-020-0527-7 PMC7611090 32483388

[B10] Baier J. Gänsbauer M. Giessler C. Arnold H. Muske M. Schleicher U. (2020). Arginase Impedes the Resolution of Colitis by Altering the Microbiome and Metabolome. J. Clin. Invest. 130 (11), 5703–5720. 10.1172/JCI126923 32721946 PMC7598089

[B11] Bakhoum S. F. Ngo B. Laughney A. M. Cavallo J.-A. Murphy C. J. Ly P. (2018). Chromosomal Instability Drives Metastasis through a Cytosolic DNA Response. Nature 553 (7689), 467–472. 10.1038/nature25432 29342134 PMC5785464

[B12] Bao S. Hu T. Liu J. Su J. Sun J. Ming Y. (2021). Genomic Instability-Derived Plasma Extracellular Vesicle-microRNA Signature as a Minimally Invasive Predictor of Risk and Unfavorable Prognosis in Breast Cancer. J. Nanobiotechnol 19 (1), 22. 10.1186/s12951-020-00767-3 PMC7802300 33436002

[B13] Barnett K. C. Coronas-Serna J. M. Zhou W. Ernandes M. J. Cao A. Kranzusch P. J. (2019). Phosphoinositide Interactions Position cGAS at the Plasma Membrane to Ensure Efficient Distinction between Self- and Viral DNA. Cell 176 (6), 1432–1446. e1411. 10.1016/j.cell.2019.01.049 30827685 PMC6697112

[B14] Bertero T. Oldham W. M. Grasset E. M. Bourget I. Boulter E. Pisano S. (2019). Tumor-Stroma Mechanics Coordinate Amino Acid Availability to Sustain Tumor Growth and Malignancy. Cel Metab. 29 (1), 124–140. 10.1016/j.cmet.2018.09.012 PMC6432652 30293773

[B15] Bhattacharya S. Srinivasan K. Abdisalaam S. Su F. Raj P. Dozmorov I. (2017). RAD51 Interconnects between DNA Replication, DNA Repair and Immunity. Nucleic Acids Res. 45 (8), 4590–4605. 10.1093/nar/gkx126 28334891 PMC5416901

[B16] Bose S. Allen A. E. Locasale J. W. (2020). The Molecular Link from Diet to Cancer Cell Metabolism. Mol. Cel. 78 (6), 1034–1044. 10.1016/j.molcel.2020.05.018 PMC7305994 32504556

[B17] Boutard V. Havouis R. Fouqueray B. Philippe C. Moulinoux J. P. Baud L. (1995). Transforming Growth Factor-Beta Stimulates Arginase Activity in Macrophages. Implications for the Regulation of Macrophage Cytotoxicity. J. Immunol. 155 (4), 2077–2084. 7636258

[B18] Boyer J. A. Spangler C. J. Strauss J. D. Cesmat A. P. Liu P. McGinty R. K. (2020). Structural Basis of Nucleosome-dependent cGAS Inhibition. Science 370 (6515), 450–454. 10.1126/science.abd0609 32913000 PMC8189757

[B19] Carriche G. M. Almeida L. Stüve P. Velasquez L. Dhillon-LaBrooy A. Roy U. (2021). Regulating T-Cell Differentiation through the Polyamine Spermidine. J. Allergy Clin. Immunol. 147 (1), 335–348. 10.1016/j.jaci.2020.04.037 32407834

[B20] Cerboni S. Jeremiah N. Gentili M. Gehrmann U. Conrad C. Stolzenberg M.-C. (2017). Intrinsic Antiproliferative Activity of the Innate Sensor STING in T Lymphocytes. J. Exp. Med. 214 (6), 1769–1785. 10.1084/jem.20161674 28484079 PMC5461003

[B21] Chan-Seng-Yue M. Kim J. C. Wilson G. W. Ng K. Figueroa E. F. O’Kane G. M. (2020). Transcription Phenotypes of Pancreatic Cancer Are Driven by Genomic Events during Tumor Evolution. Nat. Genet. 52 (2), 231–240. 10.1038/s41588-019-0566-9 31932696

[B22] Chen D. Zhang X. Li Z. Zhu B. (2021). Metabolic Regulatory Crosstalk between Tumor Microenvironment and Tumor-Associated Macrophages. Theranostics 11 (3), 1016–1030. 10.7150/thno.51777 33391518 PMC7738889

[B23] Chen Q. Boire A. Jin X. Valiente M. Er E. E. Lopez-Soto A. (2016a). Carcinoma-astrocyte gap Junctions Promote Brain Metastasis by cGAMP Transfer. Nature 533 (7604), 493–498. 10.1038/nature18268 27225120 PMC5021195

[B24] Chen Q. Sun L. Chen Z. J. (2016b). Regulation and Function of the cGAS-STING Pathway of Cytosolic DNA Sensing. Nat. Immunol. 17 (10), 1142–1149. 10.1038/ni.3558 27648547

[B25] Cheng A. N. Cheng L.-C. Kuo C.-L. Lo Y. K. Chou H.-Y. Chen C.-H. (2020). Mitochondrial Lon-Induced mtDNA Leakage Contributes to PD-L1-Mediated Immunoescape via STING-IFN Signaling and Extracellular Vesicles. J. Immunother. Cancer 8 (2), e001372. 10.1136/jitc-2020-001372 33268351 PMC7713199

[B26] Cohen D. Melamed S. Millman A. Shulman G. Oppenheimer-Shaanan Y. Kacen A. (2019). Cyclic GMP-AMP Signalling Protects Bacteria against Viral Infection. Nature 574 (7780), 691–695. 10.1038/s41586-019-1605-5 31533127

[B27] Corrales L. McWhirter S. M. Dubensky T. W. Jr. Gajewski T. F. (2016). The Host STING Pathway at the Interface of Cancer and Immunity. J. Clin. Invest. 126 (7), 2404–2411. 10.1172/JCI86892 27367184 PMC4922692

[B28] Curran E. Chen X. Corrales L. Kline D. E. Dubensky T. W. Jr. Duttagupta P. (2016). STING Pathway Activation Stimulates Potent Immunity against Acute Myeloid Leukemia. Cel Rep. 15 (11), 2357–2366. 10.1016/j.celrep.2016.05.023 PMC5116809 27264175

[B29] de Oliveira Mann C. C. Orzalli M. H. King D. S. Kagan J. C. Lee A. S. Y. Kranzusch P. J. (2019). Modular Architecture of the STING C-Terminal Tail Allows Interferon and NF-Κb Signaling Adaptation. Cel Rep. 27 (4), 1165–1175. e1165. 10.1016/j.celrep.2019.03.098 PMC7733315 31018131

[B30] Demaria O. De Gassart A. Coso S. Gestermann N. Di Domizio J. Flatz L. (2015). STING Activation of Tumor Endothelial Cells Initiates Spontaneous and Therapeutic Antitumor Immunity. Proc. Natl. Acad. Sci. USA 112 (50), 15408–15413. 10.1073/pnas.1512832112 26607445 PMC4687570

[B31] Deng L. Liang H. Xu M. Yang X. Burnette B. Arina A. (2014). STING-dependent Cytosolic DNA Sensing Promotes Radiation-Induced Type I Interferon-dependent Antitumor Immunity in Immunogenic Tumors. Immunity 41 (5), 843–852. 10.1016/j.immuni.2014.10.019 25517616 PMC5155593

[B32] Diamond M. S. Kinder M. Matsushita H. Mashayekhi M. Dunn G. P. Archambault J. M. (2011). Type I Interferon Is Selectively Required by Dendritic Cells for Immune Rejection of Tumors. J. Exp. Med. 208 (10), 1989–2003. 10.1084/jem.20101158 21930769 PMC3182061

[B33] Dillon M. T. Bergerhoff K. F. Pedersen M. Whittock H. Crespo-Rodriguez E. Patin E. C. (2019). ATR Inhibition Potentiates the Radiation-Induced Inflammatory Tumor Microenvironment. Clin. Cancer Res. 25 (11), 3392–3403. 10.1158/1078-0432.CCR-18-1821 30770349 PMC6551222

[B34] Dorta-Estremera S. Hegde V. L. Slay R. B. Sun R. Yanamandra A. V. Nicholas C. (2019). Targeting Interferon Signaling and CTLA-4 Enhance the Therapeutic Efficacy of Anti-PD-1 Immunotherapy in Preclinical Model of HPV+ Oral Cancer. J. Immunotherapy Cancer 7 (1), 252. 10.1186/s40425-019-0728-4 PMC6749627 31533840

[B35] Dou Z. Ghosh K. Vizioli M. G. Zhu J. Sen P. Wangensteen K. J. (2017). Cytoplasmic Chromatin Triggers Inflammation in Senescence and Cancer. Nature 550 (7676), 402–406. 10.1038/nature24050 28976970 PMC5850938

[B36] Driskell R. R. Watt F. M. (2015). Understanding Fibroblast Heterogeneity in the Skin. Trends Cell Biology 25 (2), 92–99. 10.1016/j.tcb.2014.10.001 25455110

[B37] Du J. Liu A. Zhu R. Zhou C. Su H. Xie G. (2019). The Different Effects of IFN-β and IFN-γ on the Tumor-Suppressive Activity of Human Amniotic Fluid-Derived Mesenchymal Stem Cells. Stem Cell Int. 2019, 1–15. 10.1155/2019/4592701 PMC6501177 31149015

[B38] Du M. Chen Z. J. (2018). DNA-induced Liquid Phase Condensation of cGAS Activates Innate Immune Signaling. Science 361 (6403), 704–709. 10.1126/science.aat1022 29976794 PMC9417938

[B39] Fang R. Wang C. Jiang Q. Lv M. Gao P. Yu X. (2017). NEMO-IKKβ Are Essential for IRF3 and NF-Κb Activation in the cGAS-STING Pathway. J.I. 199 (9), 3222–3233. 10.4049/jimmunol.1700699 28939760

[B40] Fultang L. Booth S. Yogev O. Martins da Costa B. Tubb V. Panetti S. (2020). Metabolic Engineering against the Arginine Microenvironment Enhances CAR-T Cell Proliferation and Therapeutic Activity. Blood 136 (10), 1155–1160. 10.1182/blood.2019004500 32573723 PMC7565134

[B41] Gajewski T. F. Corrales L. (2015). New Perspectives on Type I IFNs in Cancer. Cytokine Growth Factor. Rev. 26 (2), 175–178. 10.1016/j.cytogfr.2015.01.001 25630967 PMC4387009

[B42] Garber K. (2018). A New Cancer Immunotherapy Suffers a Setback. Science 360 (6389), 588. 10.1126/science.360.6389.588 29748264

[B43] Gentili M. Lahaye X. Nadalin F. Nader G. P. F. Lombardi E. P. Herve S. (2019). The N-Terminal Domain of cGAS Determines Preferential Association with Centromeric DNA and Innate Immune Activation in the Nucleus. Cel Rep. 26 (13), 3798. 10.1016/j.celrep.2019.03.049 PMC6444014 30917330

[B44] Ghaffari A. Peterson N. Khalaj K. Vitkin N. Robinson A. Francis J.-A. (2018). STING Agonist Therapy in Combination with PD-1 Immune Checkpoint Blockade Enhances Response to Carboplatin Chemotherapy in High-Grade Serous Ovarian Cancer. Br. J. Cancer 119 (4), 440–449. 10.1038/s41416-018-0188-5 30046165 PMC6133940

[B45] Grabosch S. Bulatovic M. Zeng F. Ma T. Zhang L. Ross M. (2019). Cisplatin-induced Immune Modulation in Ovarian Cancer Mouse Models with Distinct Inflammation Profiles. Oncogene 38 (13), 2380–2393. 10.1038/s41388-018-0581-9 30518877 PMC6440870

[B46] Greten F. R. Grivennikov S. I. (2019). Inflammation and Cancer: Triggers, Mechanisms, and Consequences. Immunity 51 (1), 27–41. 10.1016/j.immuni.2019.06.025 31315034 PMC6831096

[B47] Gulen M. F. Koch U. Haag S. M. Schuler F. Apetoh L. Villunger A. (2017). Signalling Strength Determines Proapoptotic Functions of STING. Nat. Commun. 8 (1), 427. 10.1038/s41467-017-00573-w 28874664 PMC5585373

[B48] Hahn W. O. Butler N. S. Lindner S. E. Akilesh H. M. Sather D. N. Kappe S. H. I. (2018). cGAS-mediated Control of Blood-Stage Malaria Promotes Plasmodium-specific Germinal center Responses. JCI insight 3 (2). 10.1172/jci.insight.94142 PMC5821207 29367469

[B49] Hartl C. A. Bertschi A. Puerto R. B. Andresen C. Cheney E. M. Mittendorf E. A. (2019). Combination Therapy Targeting Both Innate and Adaptive Immunity Improves Survival in a Pre-clinical Model of Ovarian Cancer. J. Immunotherapy Cancer 7 (1), 199. 10.1186/s40425-019-0654-5 PMC6668091 31362778

[B50] Herzner A.-M. Hagmann C. A. Goldeck M. Wolter S. Kübler K. Wittmann S. (2015). Sequence-specific Activation of the DNA Sensor cGAS by Y-form DNA Structures as Found in Primary HIV-1 cDNA. Nat. Immunol. 16 (10), 1025–1033. 10.1038/ni.3267 26343537 PMC4669199

[B51] Hinshaw D. C. Shevde L. A. (2019). The Tumor Microenvironment Innately Modulates Cancer Progression. Cancer Res. 79 (18), 4557–4566. 10.1158/0008-5472.CAN-18-3962 31350295 PMC6744958

[B52] Hintzsche H. Hemmann U. Poth A. Utesch D. Lott J. Stopper H. (2017). Fate of Micronuclei and Micronucleated Cells. Mutat. Research/Reviews Mutat. Res. 771, 85–98. 10.1016/j.mrrev.2017.02.002 28342454

[B53] Höglander E. K. Nord S. Wedge D. C. Lingjærde O. C. Silwal-Pandit L. Gythfeldt H. v. (2018). Time Series Analysis of Neoadjuvant Chemotherapy and Bevacizumab-Treated Breast Carcinomas Reveals a Systemic Shift in Genomic Aberrations. Genome Med. 10 (1), 92. 10.1186/s13073-018-0601-y 30497530 PMC6262977

[B54] Hopfner K.-P. Hornung V. (2020). Molecular Mechanisms and Cellular Functions of cGAS-STING Signalling. Nat. Rev. Mol. Cel Biol 21 (9), 501–521. 10.1038/s41580-020-0244-x 32424334

[B55] Hou P.-p. Luo L.-j. Chen H.-z. Chen Q.-t. Bian X.-l. Wu S.-f. (2020). Ectosomal PKM2 Promotes HCC by Inducing Macrophage Differentiation and Remodeling the Tumor Microenvironment. Mol. Cel. 78 (6), 1192–1206. 10.1016/j.molcel.2020.05.004 32470318

[B56] Hu M. Zhou M. Bao X. Pan D. Jiao M. Liu X. (2021). ATM Inhibition Enhances Cancer Immunotherapy by Promoting mtDNA Leakage and cGAS/STING Activation. J. Clin. Invest. 131 (3). 10.1172/JCI139333 PMC7843232 33290271

[B57] Hutton C. Heider F. Blanco-Gomez A. Banyard A. Kononov A. Zhang X. (2021). Single-cell Analysis Defines a Pancreatic Fibroblast Lineage that Supports Anti-tumor Immunity. Cancer cell 39 (9), 1227–1244. 10.1016/j.ccell.2021.06.017 34297917 PMC8443274

[B58] Jablonska J. Leschner S. Westphal K. Lienenklaus S. Weiss S. (2010). Neutrophils Responsive to Endogenous IFN-β Regulate Tumor Angiogenesis and Growth in a Mouse Tumor Model. J. Clin. Invest. 120 (4), 1151–1164. 10.1172/JCI37223 20237412 PMC2846036

[B59] Jacquelot N. Yamazaki T. Roberti M. P. Duong C. P. M. Andrews M. C. Verlingue L. (2019). Sustained Type I Interferon Signaling as a Mechanism of Resistance to PD-1 Blockade. Cell Res 29 (10), 846–861. 10.1038/s41422-019-0224-x 31481761 PMC6796942

[B60] Ji L. Zhao X. Zhang B. Kang L. Song W. Zhao B. (2019). Slc6a8-Mediated Creatine Uptake and Accumulation Reprogram Macrophage Polarization via Regulating Cytokine Responses. Immunity 51 (2), 272–284. 10.1016/j.immuni.2019.06.007 31399282

[B61] Jiang X. Wang J. Deng X. Xiong F. Zhang S. Gong Z. (2020). The Role of Microenvironment in Tumor Angiogenesis. J. Exp. Clin. Cancer Res. 39 (1), 204. 10.1186/s13046-020-01709-5 32993787 PMC7526376

[B62] Jin R. Liu B. Yu M. Song L. Gu M. Wang Z. (2021). Profiling of DNA Damage and Repair Pathways in Small Cell Lung Cancer Reveals a Suppressive Role in the Immune Landscape. Mol. Cancer 20 (1), 130. 10.1186/s12943-021-01432-5 34620176 PMC8496044

[B63] Karmakar S. Lal G. (2021). Role of Serotonin Receptor Signaling in Cancer Cells and Anti-tumor Immunity. Theranostics 11 (11), 5296–5312. 10.7150/thno.55986 33859748 PMC8039959

[B64] Kim J. Gupta R. Blanco L. P. Yang S. Shteinfer-Kuzmine A. Wang K. (2019). VDAC Oligomers Form Mitochondrial Pores to Release mtDNA Fragments and Promote Lupus-like Disease. Science 366 (6472), 1531–1536. 10.1126/science.aav4011 31857488 PMC8325171

[B65] Kim J. H. Penson A. V. Taylor B. S. Petrini J. H. J. (2019). Nbn−Mre11 Interaction Is Required for Tumor Suppression and Genomic Integrity. Proc. Natl. Acad. Sci. USA 116 (30), 15178–15183. 10.1073/pnas.1905305116 31285322 PMC6660787

[B66] Konno H. Konno K. Barber G. N. (2013). Cyclic Dinucleotides Trigger ULK1 (ATG1) Phosphorylation of STING to Prevent Sustained Innate Immune Signaling. Cell 155 (3), 688–698. 10.1016/j.cell.2013.09.049 24119841 PMC3881181

[B67] Korherr C. Gille H. Schafer R. Koenig-Hoffmann K. Dixelius J. Egland K. A. (2006). Identification of Proangiogenic Genes and Pathways by High-Throughput Functional Genomics: TBK1 and the IRF3 Pathway. Proc. Natl. Acad. Sci. 103 (11), 4240–4245. 10.1073/pnas.0511319103 16537515 PMC1449677

[B68] Kosaka A. Ishibashi K. Nagato T. Kitamura H. Fujiwara Y. Yasuda S. (2021). CD47 Blockade Enhances the Efficacy of Intratumoral STING-Targeting Therapy by Activating Phagocytes. J. Exp. Med. 218 (11). 10.1084/jem.20200792 PMC8480673 34559187

[B69] Lahaye X. Gentili M. Silvin A. Conrad C. Picard L. Jouve M. (2018). NONO Detects the Nuclear HIV Capsid to Promote cGAS-Mediated Innate Immune Activation. Cell 175 (2), 488–501. e422. 10.1016/j.cell.2018.08.062 30270045

[B70] Lai J. Fu Y. Tian S. Huang S. Luo X. Lin L. (2021). Zebularine Elevates STING Expression and Enhances cGAMP Cancer Immunotherapy in Mice. Mol. Ther. 29 (5), 1758–1771. 10.1016/j.ymthe.2021.02.005 33571681 PMC8116609

[B71] Lamberti M. J. Mentucci F. M. Roselli E. Araya P. Rivarola V. A. Rumie Vittar N. B. (2019). Photodynamic Modulation of Type 1 Interferon Pathway on Melanoma Cells Promotes Dendritic Cell Activation. Front. Immunol. 10, 2614. 10.3389/fimmu.2019.02614 31781113 PMC6856948

[B72] Larkin B. Ilyukha V. Sorokin M. Buzdin A. Vannier E. Poltorak A. (2017). Cutting Edge: Activation of STING in T Cells Induces Type I IFN Responses and Cell Death. J.I. 199 (2), 397–402. 10.4049/jimmunol.1601999 PMC5525333 28615418

[B73] Lee S. J. Yang H. Kim W. R. Lee Y. S. Lee W. S. Kong S. J. (2021). STING Activation Normalizes the Intraperitoneal Vascular-Immune Microenvironment and Suppresses Peritoneal Carcinomatosis of colon Cancer. J. Immunother. Cancer 9 (6), e002195. 10.1136/jitc-2020-002195 34145029 PMC8215239

[B74] Lemos H. Mohamed E. Huang L. Ou R. Pacholczyk G. Arbab A. S. (2016). STING Promotes the Growth of Tumors Characterized by Low Antigenicity via IDO Activation. Cancer Res. 76 (8), 2076–2081. 10.1158/0008-5472.CAN-15-1456 26964621 PMC4873329

[B75] Li A. Yi M. Qin S. Song Y. Chu Q. Wu K. (2019). Activating cGAS-STING Pathway for the Optimal Effect of Cancer Immunotherapy. J. Hematol. Oncol. 12 (1), 35. 10.1186/s13045-019-0721-x 30935414 PMC6444510

[B76] Li F. Huangyang P. Burrows M. Guo K. Riscal R. Godfrey J. (2020). FBP1 Loss Disrupts Liver Metabolism and Promotes Tumorigenesis through a Hepatic Stellate Cell Senescence Secretome. Nat. Cel Biol 22 (6), 728–739. 10.1038/s41556-020-0511-2 PMC7286794 32367049

[B77] Li W. Lu L. Lu J. Wang X. Yang C. Jin J. (2020). cGAS-STING-mediated DNA Sensing Maintains CD8 + T Cell Stemness and Promotes Antitumor T Cell Therapy. Sci. Transl. Med. 12 (549). 10.1126/scitranslmed.aay9013 32581136

[B78] Li X.-D. Wu J. Gao D. Wang H. Sun L. Chen Z. J. (2013). Pivotal Roles of cGAS-cGAMP Signaling in Antiviral Defense and Immune Adjuvant Effects. Science 341 (6152), 1390–1394. 10.1126/science.1244040 23989956 PMC3863637

[B79] Liang D. Xiao-Feng H. Guan-Jun D. Er-Ling H. Sheng C. Ting-Ting W. (2015). Activated STING Enhances Tregs Infiltration in the HPV-Related Carcinogenesis of Tongue Squamous Cells via the C-jun/CCL22 Signal. Biochim. Biophys. Acta (Bba) - Mol. Basis Dis. 1852 (11), 2494–2503. 10.1016/j.bbadis.2015.08.011 26303640

[B80] Liang H. Deng L. Hou Y. Meng X. Huang X. Rao E. (2017). Host STING-dependent MDSC Mobilization Drives Extrinsic Radiation Resistance. Nat. Commun. 8 (1), 1736. 10.1038/s41467-017-01566-5 29170400 PMC5701019

[B81] Lim W. A. June C. H. (2017). The Principles of Engineering Immune Cells to Treat Cancer. Cell 168 (4), 724–740. 10.1016/j.cell.2017.01.016 28187291 PMC5553442

[B82] Liu H. Zhang H. Wu X. Ma D. Wu J. Wang L. (2018). Nuclear cGAS Suppresses DNA Repair and Promotes Tumorigenesis. Nature 563 (7729), 131–136. 10.1038/s41586-018-0629-6 30356214

[B83] Liu S. Cai X. Wu J. Cong Q. Chen X. Li T. (2015). Phosphorylation of Innate Immune Adaptor Proteins MAVS, STING, and TRIF Induces IRF3 Activation. Science 347 (6227), aaa2630. 10.1126/science.aaa2630 25636800

[B84] Liu W. Kim G. B. Krump N. A. Zhou Y. Riley J. L. You J. (2020). Selective Reactivation of STING Signaling to Target Merkel Cell Carcinoma. Proc. Natl. Acad. Sci. USA 117 (24), 13730–13739. 10.1073/pnas.1919690117 32482869 PMC7306767

[B85] Liu X. Zhang M. Ying S. Zhang C. Lin R. Zheng J. (2017). Genetic Alterations in Esophageal Tissues from Squamous Dysplasia to Carcinoma. Gastroenterology 153 (1), 166–177. 10.1053/j.gastro.2017.03.033 28365443

[B86] Liu Y. Liang X. Dong W. Fang Y. Lv J. Zhang T. (2018). Tumor-Repopulating Cells Induce PD-1 Expression in CD8+ T Cells by Transferring Kynurenine and AhR Activation. Cancer cell 33 (3), 480–494. 10.1016/j.ccell.2018.02.005 29533786

[B87] Liu Y. Liang X. Yin X. Lv J. Tang K. Ma J. (2017). Blockade of IDO-kynurenine-AhR Metabolic Circuitry Abrogates IFN-γ-Induced Immunologic Dormancy of Tumor-Repopulating Cells. Nat. Commun. 8, 15207. 10.1038/ncomms15207 28488695 PMC5436221

[B88] Lombardo K. A. Obradovic A. Singh A. K. Liu J. L. Joice G. Kates M. (2021). BCG Invokes superior STING ‐mediated Innate Immune Response over Radiotherapy in a Carcinogen Murine Model of Urothelial Cancer. J. Pathol. 10.1002/path.5830 PMC8738146 34731491

[B89] Luecke S. Holleufer A. Christensen M. H. Jønsson K. L. Boni G. A. Sørensen L. K. (2017). cGAS Is Activated by DNA in a Length‐dependent Manner. EMBO Rep. 18 (10), 1707–1715. 10.15252/embr.201744017 28801534 PMC5623850

[B90] Luteijn R. D. Zaver S. A. Gowen B. G. Wyman S. K. Garelis N. E. Onia L. (2019). SLC19A1 Transports Immunoreactive Cyclic Dinucleotides. Nature 573 (7774), 434–438. 10.1038/s41586-019-1553-0 31511694 PMC6785039

[B91] Lv M. Chen M. Zhang R. Zhang W. Wang C. Zhang Y. (2020). Manganese Is Critical for Antitumor Immune Responses via cGAS-STING and Improves the Efficacy of Clinical Immunotherapy. Cel Res 30 (11), 966–979. 10.1038/s41422-020-00395-4 PMC7785004 32839553

[B92] Mackenzie K. J. Carroll P. Martin C.-A. Murina O. Fluteau A. Simpson D. J. (2017). cGAS Surveillance of Micronuclei Links Genome Instability to Innate Immunity. Nature 548 (7668), 461–465. 10.1038/nature23449 28738408 PMC5870830

[B93] Marcus A. Mao A. J. Lensink-Vasan M. Wang L. Vance R. E. Raulet D. H. (2018). Tumor-Derived cGAMP Triggers a STING-Mediated Interferon Response in Non-tumor Cells to Activate the NK Cell Response. Immunity 49 (4), 754–763. e754. 10.1016/j.immuni.2018.09.016 30332631 PMC6488306

[B94] McArthur K. Whitehead L. W. Heddleston J. M. Li L. Padman B. S. Oorschot V. (2018). BAK/BAX Macropores Facilitate Mitochondrial Herniation and mtDNA Efflux during Apoptosis. Science 359 (6378). 10.1126/science.aao6047 29472455

[B95] Meric-Bernstam F. Sweis R. F. Hodi F. S. Messersmith W. A. Andtbacka R. H. I. Ingham M. (2021). Phase I Dose-Escalation Trial of MIW815 (ADU-S100), an Intratumoral STING Agonist, in Patients with Advanced/Metastatic Solid Tumors or Lymphomas. Clin. Cancer Res., CCR–21. 10.1158/1078-0432.CCR-21-1963 34716197

[B96] Michalski S. de Oliveira Mann C. C. Stafford C. A. Witte G. Bartho J. Lammens K. (2020). Structural Basis for Sequestration and Autoinhibition of cGAS by Chromatin. Nature 587 (7835), 678–682. 10.1038/s41586-020-2748-0 32911480

[B97] Miska J. Rashidi A. Lee-Chang C. Gao P. Lopez-Rosas A. Zhang P. (2021). Polyamines Drive Myeloid Cell Survival by Buffering Intracellular pH to Promote Immunosuppression in Glioblastoma. Sci. Adv. 7 (8). 10.1126/sciadv.abc8929 PMC7888943 33597238

[B98] Mitchell T. C. Hamid O. Smith D. C. Bauer T. M. Wasser J. S. Olszanski A. J. (2018). Epacadostat Plus Pembrolizumab in Patients with Advanced Solid Tumors: Phase I Results from a Multicenter, Open-Label Phase I/II Trial (ECHO-202/KEYNOTE-037). Jco 36 (32), 3223–3230. 10.1200/JCO.2018.78.9602 PMC6225502 30265610

[B99] Nandakumar R. Tschismarov R. Meissner F. Prabakaran T. Krissanaprasit A. Farahani E. (2019). Intracellular Bacteria Engage a STING-TBK1-MVB12b Pathway to Enable Paracrine cGAS-STING Signalling. Nat. Microbiol. 4 (4), 701–713. 10.1038/s41564-019-0367-z 30804548 PMC6433288

[B100] Ohkuri T. Ghosh A. Kosaka A. Zhu J. Ikeura M. David M. (2014). STING Contributes to Antiglioma Immunity via Triggering Type I IFN Signals in the Tumor Microenvironment. Cancer Immunol. Res. 2 (12), 1199–1208. 10.1158/2326-6066.CIR-14-0099 25300859 PMC4258479

[B101] Opitz C. A. Litzenburger U. M. Sahm F. Ott M. Tritschler I. Trump S. (2011). An Endogenous Tumour-Promoting Ligand of the Human Aryl Hydrocarbon Receptor. Nature 478 (7368), 197–203. 10.1038/nature10491 21976023

[B102] Pailler E. Auger N. Lindsay C. R. Vielh P. Islas-Morris-Hernandez A. Borget I. (2015). High Level of Chromosomal Instability in Circulating Tumor Cells of ROS1-Rearranged Non-small-cell Lung Cancer. Ann. Oncol. 26 (7), 1408–1415. 10.1093/annonc/mdv165 25846554 PMC4478971

[B103] Pantelidou C. Sonzogni O. De Oliveria Taveira M. Mehta A. K. Kothari A. Wang D. (2019). PARP Inhibitor Efficacy Depends on CD8+ T-Cell Recruitment via Intratumoral STING Pathway Activation in BRCA-Deficient Models of Triple-Negative Breast Cancer. Cancer Discov. 9 (6), 722–737. 10.1158/2159-8290.CD-18-1218 31015319 PMC6548644

[B104] Qu X. Tang Y. Hua S. (2018). Immunological Approaches towards Cancer and Inflammation: A Cross Talk. Front. Immunol. 9, 563. 10.3389/fimmu.2018.00563 29662489 PMC5890100

[B105] Roci I. Watrous J. D. Lagerborg K. A. Lafranchi L. Lindqvist A. Jain M. (2019). Mapping Metabolic Events in the Cancer Cell Cycle Reveals Arginine Catabolism in the Committed SG2M Phase. Cel Rep. 26 (7), 1691–1700. 10.1016/j.celrep.2019.01.059 PMC6663478 30759381

[B106] Rodriguez P. C. Quiceno D. G. Ochoa A. C. (2007). L-arginine Availability Regulates T-Lymphocyte Cell-Cycle Progression. Blood 109 (4), 1568–1573. 10.1182/blood-2006-06-031856 17023580 PMC1794048

[B107] Ronca R. Benkheil M. Mitola S. Struyf S. Liekens S. (2017). Tumor Angiogenesis Revisited: Regulators and Clinical Implications. Med. Res. Rev. 37 (6), 1231–1274. 10.1002/med.21452 28643862

[B108] Sadelain M. Rivière I. Riddell S. (2017). Therapeutic T Cell Engineering. Nature 545 (7655), 423–431. 10.1038/nature22395 28541315 PMC5632949

[B109] Sahai E. Astsaturov I. Cukierman E. DeNardo D. G. Egeblad M. Evans R. M. (2020). A Framework for Advancing Our Understanding of Cancer-Associated Fibroblasts. Nat. Rev. Cancercancer 20 (3), 174–186. 10.1038/s41568-019-0238-1 PMC7046529 31980749

[B110] Shang G. Zhang C. Chen Z. J. Bai X.-c. Zhang X. (2019). Cryo-EM Structures of STING Reveal its Mechanism of Activation by Cyclic GMP-AMP. Nature 567 (7748), 389–393. 10.1038/s41586-019-0998-5 30842659 PMC6859894

[B111] Shang G. Zhu D. Li N. Zhang J. Zhu C. Lu D. (2012). Crystal Structures of STING Protein Reveal Basis for Recognition of Cyclic Di-GMP. Nat. Struct. Mol. Biol. 19 (7), 725–727. 10.1038/nsmb.2332 22728660

[B112] Shi J. Chen C. Ju R. Wang Q. Li J. Guo L. (2019). Carboxyamidotriazole Combined with IDO1-Kyn-AhR Pathway Inhibitors Profoundly Enhances Cancer Immunotherapy. J. Immunotherapy Cancer 7 (1), 246. 10.1186/s40425-019-0725-7 PMC6740021 31511064

[B113] Sivick K. E. Desbien A. L. Glickman L. H. Reiner G. L. Corrales L. Surh N. H. (2018). Magnitude of Therapeutic STING Activation Determines CD8+ T Cell-Mediated Anti-tumor Immunity. Cel Rep. 25 (11), 3074–3085. 10.1016/j.celrep.2018.11.047 30540940

[B114] Smith T. T. Moffett H. F. Stephan S. B. Opel C. F. Dumigan A. G. Jiang X. (2017). Biopolymers Codelivering Engineered T Cells and STING Agonists Can Eliminate Heterogeneous Tumors. J. Clin. Invest. 127 (6), 2176–2191. 10.1172/JCI87624 28436934 PMC5451231

[B115] Song Z.-M. Lin H. Yi X.-M. Guo W. Hu M.-M. Shu H.-B. (2020). KAT5 Acetylates cGAS to Promote Innate Immune Response to DNA Virus. Proc. Natl. Acad. Sci. USA 117 (35), 21568–21575. 10.1073/pnas.1922330117 32817552 PMC7474609

[B116] Sun L. Wu J. Du F. Chen X. Chen Z. J. (2013). Cyclic GMP-AMP Synthase Is a Cytosolic DNA Sensor that Activates the Type I Interferon Pathway. Science 339 (6121), 786–791. 10.1126/science.1232458 23258413 PMC3863629

[B117] Takano S. Ishikawa E. Matsuda M. Yamamoto T. Matsumura A. (2014). Interferon-β Inhibits Glioma Angiogenesis through Downregulation of Vascular Endothelial Growth Factor and Upregulation of Interferon Inducible Protein 10. Int. J. Oncol. 45 (5), 1837–1846. 10.3892/ijo.2014.2620 25175315 PMC4203325

[B118] Takenaka M. C. Gabriely G. Rothhammer V. Mascanfroni I. D. Wheeler M. A. Chao C.-C. (2019). Control of Tumor-Associated Macrophages and T Cells in Glioblastoma via AHR and CD39. Nat. Neurosci. 22 (5), 729–740. 10.1038/s41593-019-0370-y 30962630 PMC8052632

[B119] Tang C.-H. A. Zundell J. A. Ranatunga S. Lin C. Nefedova Y. Del Valle J. R. (2016). Agonist-Mediated Activation of STING Induces Apoptosis in Malignant B Cells. Cancer Res. 76 (8), 2137–2152. 10.1158/0008-5472.CAN-15-1885 26951929 PMC4873432

[B120] Tang L. Wang Z. Mu Q. Yu Z. Jacobson O. Li L. (2020). Targeting Neutrophils for Enhanced Cancer Theranostics. Adv. Mater. 32 (33), 2002739. 10.1002/adma.202002739 32656801

[B121] Tao J. Zhou X. Jiang Z. (2016). cGAS-cGAMP-STING: The Three Musketeers of Cytosolic DNA Sensing and Signaling. IUBMB life 68 (11), 858–870. 10.1002/iub.1566 27706894

[B122] Telli M. L. Timms K. M. Reid J. Hennessy B. Mills G. B. Jensen K. C. (2016). Homologous Recombination Deficiency (HRD) Score Predicts Response to Platinum-Containing Neoadjuvant Chemotherapy in Patients with Triple-Negative Breast Cancer. Clin. Cancer Res. 22 (15), 3764–3773. 10.1158/1078-0432.CCR-15-2477 26957554 PMC6773427

[B123] Vanpouille-Box C. Demaria S. Formenti S. C. Galluzzi L. (2018). Cytosolic DNA Sensing in Organismal Tumor Control. Cancer cell 34 (3), 361–378. 10.1016/j.ccell.2018.05.013 30216189

[B124] Volkman H. E. Cambier S. Gray E. E. Stetson D. B. (2019). Tight Nuclear Tethering of cGAS Is Essential for Preventing Autoreactivity. eLife 8. 10.7554/eLife.47491 PMC6927687 31808743

[B125] Wang C. Guan Y. Lv M. Zhang R. Guo Z. Wei X. (2018). Manganese Increases the Sensitivity of the cGAS-STING Pathway for Double-Stranded DNA and Is Required for the Host Defense against DNA Viruses. Immunity 48 (4), 675–687. 10.1016/j.immuni.2018.03.017 29653696

[B126] Wang H. Hu S. Chen X. Shi H. Chen C. Sun L. (2017). cGAS Is Essential for the Antitumor Effect of Immune Checkpoint Blockade. Proc. Natl. Acad. Sci. USA 114 (7), 1637–1642. 10.1073/pnas.1621363114 28137885 PMC5320994

[B127] Wang Y. Luo J. Alu A. Han X. Wei Y. Wei X. (2020). cGAS-STING Pathway in Cancer Biotherapy. Mol. Cancer 19 (1), 136. 10.1186/s12943-020-01247-w 32887628 PMC7472700

[B128] Wang Z. Chen J. Hu J. Zhang H. Xu F. He W. (2019). cGAS/STING axis Mediates a Topoisomerase II Inhibitor-Induced Tumor Immunogenicity. J. Clin. Invest. 129 (11), 4850–4862. 10.1172/JCI127471 31408442 PMC6819145

[B129] Weiner G. J. (2009). CpG Oligodeoxynucleotide-Based Therapy of Lymphoid Malignancies. Adv. Drug Deliv. Rev. 61 (3), 263–267. 10.1016/j.addr.2008.12.006 19168102

[B130] Weiss J. M. Guérin M. V. Regnier F. Renault G. Galy-Fauroux I. Vimeux L. (2017). The STING Agonist DMXAA Triggers a Cooperation between T Lymphocytes and Myeloid Cells that Leads to Tumor Regression. Oncoimmunology 6 (10), e1346765. 10.1080/2162402X.2017.1346765 29123960 PMC5665074

[B131] White M. J. McArthur K. Metcalf D. Lane R. M. Cambier J. C. Herold M. J. (2014). Apoptotic Caspases Suppress mtDNA-Induced STING-Mediated Type I IFN Production. Cell 159 (7), 1549–1562. 10.1016/j.cell.2014.11.036 25525874 PMC4520319

[B132] Winkler J. Abisoye-Ogunniyan A. Metcalf K. J. Werb Z. (2020). Concepts of Extracellular Matrix Remodelling in Tumour Progression and Metastasis. Nat. Commun. 11 (1), 5120. 10.1038/s41467-020-18794-x 33037194 PMC7547708

[B133] Woo S.-R. Fuertes M. B. Corrales L. Spranger S. Furdyna M. J. Leung M. Y. K. (2014). STING-dependent Cytosolic DNA Sensing Mediates Innate Immune Recognition of Immunogenic Tumors. Immunity 41 (5), 830–842. 10.1016/j.immuni.2014.10.017 25517615 PMC4384884

[B134] Wu M.-Z. Cheng W.-C. Chen S.-F. Nieh S. O’Connor C. Liu C.-L. (2017). miR-25/93 Mediates Hypoxia-Induced Immunosuppression by Repressing cGAS. Nat. Cel Biol 19 (10), 1286–1296. 10.1038/ncb3615 PMC5658024 28920955

[B135] Xia T. Konno H. Ahn J. Barber G. N. (2016a). Deregulation of STING Signaling in Colorectal Carcinoma Constrains DNA Damage Responses and Correlates with Tumorigenesis. Cel Rep. 14 (2), 282–297. 10.1016/j.celrep.2015.12.029 PMC4845097 26748708

[B136] Xia T. Konno H. Barber G. N. (2016b). Recurrent Loss of STING Signaling in Melanoma Correlates with Susceptibility to Viral Oncolysis. Cancer Res. 76 (22), 6747–6759. 10.1158/0008-5472.CAN-16-1404 27680683

[B137] Xu M. M. Pu Y. Han D. Shi Y. Cao X. Liang H. (2017). Dendritic Cells but Not Macrophages Sense Tumor Mitochondrial DNA for Cross-Priming through Signal Regulatory Protein α Signaling. Immunity 47 (2), 363–373. 10.1016/j.immuni.2017.07.016 28801234 PMC5564225

[B138] Xu N. Palmer D. C. Robeson A. C. Shou P. Bommiasamy H. Laurie S. J. (2021). STING Agonist Promotes CAR T Cell Trafficking and Persistence in Breast Cancer. J. Exp. Med. 218 (2). 10.1084/jem.20200844 PMC7780733 33382402

[B139] Yang H. Lee W. S. Kong S. J. Kim C. G. Kim J. H. Chang S. K. (2019). STING Activation Reprograms Tumor Vasculatures and Synergizes with VEGFR2 Blockade. J. Clin. Invest. 129 (10), 4350–4364. 10.1172/JCI125413 31343989 PMC6763266

[B140] Yeh Y.-H. Hsiao H.-F. Yeh Y.-C. Chen T.-W. Li T.-K. (2018). Inflammatory Interferon Activates HIF-1α-Mediated Epithelial-To-Mesenchymal Transition via PI3K/AKT/mTOR Pathway. J. Exp. Clin. Cancer Res. 37 (1), 70. 10.1186/s13046-018-0730-6 29587825 PMC5870508

[B141] Yu C.-H. Davidson S. Harapas C. R. Hilton J. B. Mlodzianoski M. J. Laohamonthonkul P. (2020). TDP-43 Triggers Mitochondrial DNA Release via mPTP to Activate cGAS/STING in ALS. Cell 183 (3), 636–649. 10.1016/j.cell.2020.09.020 33031745 PMC7599077

[B142] Yuan L. Mao Y. Luo W. Wu W. Xu H. Wang X. L. (2017). Palmitic Acid Dysregulates the Hippo-YAP Pathway and Inhibits Angiogenesis by Inducing Mitochondrial Damage and Activating the Cytosolic DNA Sensor cGAS-STING-IRF3 Signaling Mechanism. J. Biol. Chem. 292 (36), 15002–15015. 10.1074/jbc.M117.804005 28698384 PMC5592676

[B143] Zhang C. Shang G. Gui X. Zhang X. Bai X.-c. Chen Z. J. (2019). Structural Basis of STING Binding with and Phosphorylation by TBK1. Nature 567 (7748), 394–398. 10.1038/s41586-019-1000-2 30842653 PMC6862768

[B144] Zhang X. Bai X.-c. Chen Z. J. (2020). Structures and Mechanisms in the cGAS-STING Innate Immunity Pathway. Immunity 53 (1), 43–53. 10.1016/j.immuni.2020.05.013 32668227

[B145] Zhang X. Shi H. Wu J. Zhang X. Sun L. Chen C. (2013). Cyclic GMP-AMP Containing Mixed Phosphodiester Linkages Is an Endogenous High-Affinity Ligand for STING. Mol. Cel. 51 (2), 226–235. 10.1016/j.molcel.2013.05.022 PMC3808999 23747010

[B146] Zhang Y. Recouvreux M. V. Jung M. Galenkamp K. M. O. Li Y. Zagnitko O. (2021). Macropinocytosis in Cancer-Associated Fibroblasts Is Dependent on CaMKK2/ARHGEF2 Signaling and Functions to Support Tumor and Stromal Cell Fitness. Cancer Discov. 11 (7), 1808–1825. 10.1158/2159-8290.CD-20-0119 33653692 PMC8292164

[B147] Zhao B. Du F. Xu P. Shu C. Sankaran B. Bell S. L. (2019). A Conserved PLPLRT/SD Motif of STING Mediates the Recruitment and Activation of TBK1. Nature 569 (7758), 718–722. 10.1038/s41586-019-1228-x 31118511 PMC6596994

[B148] Zhou C. Chen X. Planells-Cases R. Chu J. Wang L. Cao L. (2020). Transfer of cGAMP into Bystander Cells via LRRC8 Volume-Regulated Anion Channels Augments STING-Mediated Interferon Responses and Anti-viral Immunity. Immunity 52 (5), 767–781. e766. 10.1016/j.immuni.2020.03.016 32277911

[B149] Zhou W. Whiteley A. T. de Oliveira Mann C. C. Morehouse B. R. Nowak R. P. Fischer E. S. (2018). Structure of the Human cGAS-DNA Complex Reveals Enhanced Control of Immune Surveillance. Cell 174 (2), 300–311. e311. 10.1016/j.cell.2018.06.026 30007416 PMC6084792

[B150] Zhou Y. Fei M. Zhang G. Liang W.-C. Lin W. Wu Y. (2020). Blockade of the Phagocytic Receptor MerTK on Tumor-Associated Macrophages Enhances P2X7R-dependent STING Activation by Tumor-Derived cGAMP. Immunity 52 (2), 357–373. 10.1016/j.immuni.2020.01.014 32049051

[B151] Zierhut C. Yamaguchi N. Paredes M. Luo J.-D. Carroll T. Funabiki H. (2019). The Cytoplasmic DNA Sensor cGAS Promotes Mitotic Cell Death. Cell 178 (2), 302–315. 10.1016/j.cell.2019.05.035 31299200 PMC6693521

